# Myeloperoxidase-Derived Oxidants Induce Blood-Brain Barrier Dysfunction *In Vitro* and *In Vivo*

**DOI:** 10.1371/journal.pone.0064034

**Published:** 2013-05-14

**Authors:** Andreas Üllen, Evelin Singewald, Viktoria Konya, Günter Fauler, Helga Reicher, Christoph Nusshold, Astrid Hammer, Dagmar Kratky, Akos Heinemann, Peter Holzer, Ernst Malle, Wolfgang Sattler

**Affiliations:** 1 Institute of Molecular Biology and Biochemistry, Medical University of Graz, Austria; 2 Institute of Experimental and Clinical Pharmacology, Medical University of Graz, Austria; 3 Clinical Institute of Medical and Chemical Laboratory Diagnostics, Medical University of Graz, Austria; 4 Institute of Cell Biology, Histology and Embryology, Medical University of Graz, Austria; University of California, Riverside, United States of America

## Abstract

Peripheral leukocytes can exacerbate brain damage by release of cytotoxic mediators that disrupt blood-brain barrier (BBB) function. One of the oxidants released by activated leukocytes is hypochlorous acid (HOCl) formed via the myeloperoxidase (MPO)-H_2_O_2_-Cl^−^ system. In the present study we examined the role of leukocyte activation, leukocyte-derived MPO and MPO-generated oxidants on BBB function in vitro and in vivo. In a mouse model of lipopolysaccharide (LPS)-induced systemic inflammation, neutrophils that had become adherent released MPO into the cerebrovasculature. In vivo, LPS-induced BBB dysfunction was significantly lower in MPO-deficient mice as compared to wild-type littermates. Both, fMLP-activated leukocytes and the MPO-H_2_O_2_-Cl^−^ system inflicted barrier dysfunction of primary brain microvascular endothelial cells (BMVEC) that was partially rescued with the MPO inhibitor 4-aminobenzoic acid hydrazide. BMVEC treatment with the MPO-H_2_O_2_-Cl^−^ system or activated neutrophils resulted in the formation of plasmalogen-derived chlorinated fatty aldehydes. 2-chlorohexadecanal (2-ClHDA) severely compromised BMVEC barrier function and induced morphological alterations in tight and adherens junctions. In situ perfusion of rat brain with 2-ClHDA increased BBB permeability in vivo. 2-ClHDA potently activated the MAPK cascade at physiological concentrations. An ERK1/2 and JNK antagonist (PD098059 and SP600125, respectively) protected against 2-ClHDA-induced barrier dysfunction in vitro. The current data provide evidence that interference with the MPO pathway could protect against BBB dysfunction under (neuro)inflammatory conditions.

## Introduction

The neurovascular unit physically separates most regions of the brain from the peripheral circulation to maintain the specialized micromilieu of the central nervous system [1]. Brain microvascular endothelial cells (BMVEC) constitute the morphological basis of the blood-brain barrier (BBB) by the formation of tight junction (TJ) complexes [2]. The presence of TJ prevents paracellular transport of molecules and cells and maintains brain homeostasis via elaborately regulated transport mechanisms. In addition to this ‘sealing’ function, TJ complexes physically separate a luminal and abluminal compartment thereby sustaining the polarized phenotype of BMVEC [3]. This is of particular importance since correct patterning of receptors and/or transport proteins at the apical or basolateral side of the plasma membrane maintains homeostasis for cells present at the neurovascular unit [1].

Under inflammatory conditions BBB function is compromised and can aggravate neuronal dysfunction [4]. Pathways thought to initiate BBB dysfunction include the kinin system, excitotoxicity, neutrophil recruitment/activation, dysfunctional mitochondria, NADPH oxidase or nitric oxide synthase activation, and macrophage/microglial activation, all of which converge on the formation of reactive species [5]. TJ proteins are particularly sensitive to alterations of the intracellular redox status, resulting in barrier dysfunction [6]. A strong oxidant attacking a wide range of biological targets is hypochlorous acid (HOCl) generated by the myeloperoxidase (MPO)-H_2_O_2_-Cl^−^ system of activated phagocytes such as neutrophils and monocytes [7]. HOCl can covalently modify lipids and/or proteins causing local tissue damage and amplification of the inflammatory cascade [8]. MPO can promote endothelial dysfunction, upregulate inducible nitric oxide synthase and impair lipoprotein function [9]. In multiple sclerosis (MS), MPO is present in microglia/macrophages at lesion sites [10]. It has been shown recently that pharmacological inhibition of MPO reduced the severity of clinical symptoms in a mouse model of MS [11]. Results of this study [11] suggest that the MPO inhibitor 4-aminobenzoic acid hydrazide (4-ABAH) inhibited mainly released, extracellular MPO in brains of these mice. The involvement of MPO in barrier dysfunction was also suggested in bacterial meningitis [12], [13]. On the other hand, blockade of MPO activity was shown to augment rather than inhibit rotenone-induced reactive oxygen species generation and glial cell death. In addition, rotenone-triggered neuronal injury is more pronounced in co-cultures with glial cells from MPO-deficient (MPO^−/−^) mice [14]. Extracellular MPO can result from neutrophil extracellular traps (NETs; [15]). We could demonstrate significantly elevated MPO protein levels in brains of mice that received a single, peripheral lipopolysaccharide (LPS) injection [16]. This was accompanied by a significant decrease of the brain plasmalogen concentration and concomitant formation of 2-chlorohexadecanal (2-ClHDA), a chlorinated fatty aldehyde generated from HOCl-mediated attack of plasmalogens (ether phospholipids) [16]. It is conceivable that oxidative modification of BMVEC plasmalogens might have detrimental effects on BBB function because i) plasmalogens are important constituents of lipid rafts [17], and ii) barrier and fence function of TJ complexes depend on membrane scaffolding and transporter lipid rafts [18].

During the present study we investigated the role of MPO-derived oxidants in BBB dysfunction under inflammatory conditions in vitro and in vivo. We explored the effects of activated polymorphonuclear leukocytes (PMNL) and purified MPO on barrier function of primary porcine BMVEC and studied BBB permeability in wild-type and MPO^−/−^ mice in response to peripheral LPS administration. We then quantitated the plasmalogen content of BMVEC, studied the impact of isolated MPO and activated PMNL on chloro fatty aldehyde formation, and examined the effects of 2-ClHDA on barrier function in vitro and in vivo.

## Methods

LPS from *Escherichia coli* (0111:B4), pentobarbital sodium salt, heparin sodium salt, Evans Blue (EB), sodium fluorescein (SF), bovine serum albumin (BSA), DMEM Ham’s F12, hydrocortisone (HC), sodium hypochlorite (NaOCl), H_2_O_2_, methionine, the MPO inhibitor 4-aminobenzoic acid hydrazide (4-ABAH), N,N-dimethyl formamide (DMF), dimethyl sulfoxid (DMSO), phenylmethylsulfonyl fluoride (PMSF), aprotinin, leupeptin, pepstatin and other protease inhibitors were from Sigma Aldrich (Vienna, Austria). Earl’s medium M199, penicillin, streptomycin, glutamine, gentamycin, and trypsin were from Gibco (Vienna, Austria). Ox serum was from PAA Laboratories (Linz, Austria). Plastic ware for cell culture was obtained from Costar (Vienna, Austria) or VWR (Vienna, Austria). Lab-Tek® chamber slides were from Bartelt (Graz, Austria). ECIS electrode arrays (8W10E+) were from Ibidi (Martinsried, Germany). N-Formylmethionyl-leucyl-phenylalanine (fMLP) and PD098059 were from Calbiochem (La Jolla, CA). SP600125 and SB203580 were from Merck (Darmstadt, Germany). Human recombinant TNFα was from Invitrogen (Vienna, Austria). MPO was from Planta Naturstoffe (Vienna, Austria). Polyvinylidene difluoride transfer membrane (Biotrace™ PVDF) was from Pall Corporation (Vienna, Austria). Phosphospecific rabbit polyclonal anti-p38 MAPK (Tyr180/Tyr182; pp38), anti-SAPK/JNK (Thr183/Tyr185, pJNK1/2), pan-specific rabbit monoclonal anti-p42/44 MAPK (ERK1/2), mouse monoclonal anti-p38 MAPK and rabbit polyclonal anti-SAPK/JNK1/2 antibodies were from Cell Signaling Technology (Beverly, MA). Horseradish peroxidase (HRP)-labeled secondary goat anti-rabbit IgG was from Pierce (Rockford, IL) and HRP-labeled secondary goat anti-mouse IgG was from Santa Cruz Biotechnology (Dallas, TX). Polyclonal rabbit anti-MPO and polyclonal rabbit anti-von Willebrand factor (vWF) were from Dako (Vienna, Austria). Fluorescein isothiocyanate-labeled monoclonal rat anti-neutophil antibody was from Abcam (Cambridge, UK), polyclonal rabbit anti-Zonula occludens-1 (ZO-1) was from Zymed (Vienna, Austria), and monoclonal mouse anti-vascular endothelial (VE)-cadherin was from Santa Cruz Biotechnology (Dallas, TX). Cyanine (Cy)-2 (goat anti-mouse IgG and goat anti-rat IgG)-, Cy-3 (goat anti-rabbit IgG)- and Cy-5 (goat anti-rabbit IgG)-labeled antibodies were from Jackson Dianova (Hamburg, Germany). ECL Western blotting substrate was from Pierce (Rockford, IL). ECL Plus™ Western blotting detection reagents were from GE Healthcare (Vienna, Austria) and CURIX Ultra UV-G X-ray films were from Agfa (Mortsel, Belgium). DyLight 650 Microscale Antibody Labeling Kit was from Thermo Fisher Scientific (Rockford, IL). Ultra V Blocking solution and AEC Substrate System was from Lab Vision Corp. (Fermont, CA). Mayer's hemalum and Kaiser's glycerol gelatin was from Merck (Vienna, Austria). Antibody diluent for immunohistochemistry was from Dako (Vienna, Austria), and Moviol was from Calbiochem-Novabiochem (La Jolla, CA).

### Animals and Animal Experiments

Male C57BL/6 mice (8–10 weeks, 20–30 g) and male Sprague-Dawley rats (250–300 g) were obtained from the Institut für Versuchstierkunde (Himberg, Austria). MPO^−/−^ mice (B6.129X1-Mpo^tm1Lus^/J) were from the Jackson Laboratory (Bar Harbor, ME). Mating was performed between homozygotes and genotyping was performed from tail tips using standard PCR. All animal experiments were performed in accordance with animal care ethics approval and guidelines, as per Animal Care Certificate BMWF-66.010/0055-II/3b/2011 of the Austrian Federal Ministry of Science and Research (BMWF, Vienna, Austria). All animals were kept on a 12 h light/dark cycle with free access to food and water. Systemic inflammation was induced by i.p. injection of a single dose of LPS (250 µg/30 g body weight).

### Immunohistochemistry and Triple Immunofluorescence in Murine Brain Cryosections

After indicated time periods of systemic inflammation mice were killed by cervical dislocation, brains were removed, snap frozen in liquid N_2_, and stored at −70°C or immediately processed for analyses. Serial sagittal cryosections (5 µm) were collected on glass slides, air dried for 2 h at room temperature (RT), fixed in acetone for 5 min at RT and stored at −70°C until required. For triple immunofluorescence rabbit anti-vWF was DyLight 650-labeled according to the manufacturer’s recommendations. Prior to immunostaining, sections were thawed and air-dried for 30 min at RT followed by fixation in acetone for 5 min at RT. After re-hydration in PBS, sections were blocked with UV ultra block for 10 min. For immunohistochemical studies sections were incubated with rabbit anti-human MPO antiserum (1∶500) and HRP-labeled goat anti-rabbit IgG (1∶200) each for 30 min. After 3-amino-9-ethylcarbazole (AEC) development and termination by washing with distilled water according to the manufacturer’s recommendations (Lab Vision AEC Substrate System) the sections were counterstained with Mayer's hemalum and mounted with Kaiser's glycerol gelatin. Analysis of sections was performed with a Leica DM600B microscope equipped with an Olympus DP72 digital camera (Leica, Heidelberg, Germany). For triple immunofluorescence, the sections were sequentially incubated for 30 min at RT with primary antibodies (rabbit anti-human MPO, 1∶500, and rat anti-mouse neutrophils, 1∶50) and corresponding secondary antibodies (Cy-3 labeled goat anti-rabbit IgG, Cy-2 labeled goat anti-rat IgG; dilutions 1∶300; Cy-2 labeling was used to enhance FITC-signal), followed by a blocking step with rabbit non-immune IgG (1∶25) and incubation (30 min) with DyLight650 labeled vWF (1∶75). Immunofluorescence stained sections were mounted with Moviol and analyzed on a confocal laser-scanning microscope (Leica SP2) using 488 nm for excitation of Cy-2, 543 nm for Cy-3 and 647 nm for Dylight 650. Detected emission wavelengths were 500 to 535 nm for Cy-2, 555 nm to 620 nm for Cy-3, and 665 nm to 750 nm for Dylight 650. All incubations were performed in a moist chamber at RT in the dark (for immunofluorescence) and PBS was used for three consecutive washing steps (each 5 min) in between.

### Isolation and Culture of Primary Porcine Brain Microvascular Endothelial Cells (BMVEC)

BMVEC from porcine brains were isolated by a combination of mechanical disintegration, enzymatic digestion, and centrifugation steps, and were cultured as described [19].

### Preparation and TNFα Priming of Human Polymorphonuclear Leukocytes (PMNL)

Blood samples were taken from healthy volunteers giving written informed consent, in accordance with a protocol approved by the Ethics Committee of the Medical University of Graz (Ethics Certificate 17–291ex05/06). PMNL (containing approx. 98% neutrophils and 2% eosinophils) were prepared as described [20] using dextran sedimentation of erythrocytes followed by centrifugation on Histopaque gradients. All separation steps were performed at RT. The resulting purity and viability of neutrophils was >95%. For assays PMNL were primed with 10 ng/ml TNFα for 15 min at RT (TNFα was removed by a subsequent washing step) followed by co-incubation with BMVEC. In view of the mixed species system used during the present study, it is important to note that human neutrophils respond in a similar manner to exposure toward human or porcine endothelial cells [21].

### Measurement of Secreted Peroxidase Activity of PMNL in the Presence of BMVEC

Primed or non-primed PMNL (2×10^6^ cells) were added to BMVEC (1.5×10^5^). After 15 min TNFα-primed or unprimed PMNL were treated with LPS (1 µg/ml), PMA (250 nM), fMLP (10 µM) or vehicle for 3 h at 37°C. Where mentioned, the MPO 4-ABAH (100 µM) was added to cells prior to activation for 3 h. Afterwards the medium containing PMNL were collected and centrifuged. The supernatants (30 µl aliquots) were tested for peroxidase activity by the addition of 2.8 mM tetramethylbenzidine (TMB) and 1 mM H_2_O_2_ in 96-well plates [22].

### Electrical Cell-substrate Impedance Sensing (ECIS)

BMVEC barrier function of HC (500 nM)-induced monolayers was investigated by impedance measurement at 4 kHz on collagen-coated gold electrodes of 8W10E+ arrays using an ECIS Z System (Applied Biophysics, Troy, NY). Experiments were performed either in medium or in HBSS (pH 6). Naïve or primed PMNL were resuspended in endothelial culture medium/HBSS and added to BMVEC monolayers. After 15 min, PMNL were stimulated with 10 µM fMLP (stock added in EtOH, final concentration of vehicle was 0.2%). MPO, H_2_O_2_, 4-ABAH, and methionine were prepared as stock solutions in HBSS. MAPK inhibitors were added in DMSO (final concentration between 0.2–0.4%). H_2_O_2_ was added 4 times every 3 min to give a final concentration of 500 µM.

### Analysis of BBB Integrity *in vivo*

Changes in vascular permeability during systemic inflammation were determined in MPO^−/−^ and wild-type mice by i.p. injection of 3% EB in PBS (120 µl/30 g body weight) at treatment start (injection of PBS or 250 µg LPS/30 g body weight). After 12 h mice were anesthetized with 150 mg/kg pentobarbital (Nembutal) and transcardially perfused with 25 ml PBS. Subsequently, brains were removed, weighed, and homogenized in DMF. After extraction for 1 h on a rotating wheel samples were centrifuged at 21,500 *g* for 15**min and EB was quantified spectrophotometrically at 620 nm using an external calibration curve.

Synthesis and negative ion chemical ionization-gas chromatography-mass spectrometry (NICI-GC-MS) analysis of fatty aldehydes (FALDs) and 2-chloro fatty aldehydes (2-ClFALDs).

2-chlorohexadecanal (2-ClHDA), 2-Cl[^13^C_8_]HDA and [^13^C_8_]HDA were synthesized and purified as described [16]. 2-ClHDA was prepared as stock solution in DMSO (final concentration in culture medium or Ringer solution was 0.2–0.4%; (v/v)). The acid lability of the vinyl ether bond of plasmalogens was utilized to hydrolyze FALDs from plasmalogens and subject them to further GC-MS analysis [23]. To quantify MPO-dependent plasmalogen modification BMVEC (9.2×10^5^ cells) were washed twice with HBSS (pH 6) and incubated in the presence of MPO (120 nM final concentration) and 1 µg 2-Cl[^13^C_8_]HDA at 37°C under mild shaking. The reaction was immediately started by the addition of H_2_O_2_ (500 µM final concentration; four additions of 125 µM H_2_O_2_ at 3 min intervals). Subsequently, BMVEC modification was allowed to proceed at 37°C for 2 h. For quantification of neutrophil-dependent plasmalogen modification BMVEC (9.2×10^5^ cells) were washed twice with HBSS (pH 6) and incubated in the presence of PMNL (5×10^6^). After addition of 250 ng 2-Cl[^13^C_8_]HDA PMNL were stimulated with PMA (250 nM), or primed with TNFα and stimulated with LPS (1 µg/ml) or fMLP (10 µM) for 3 h. Total FALDs were quantified after acidic hydrolysis of BMVEC in 1 M HCl (2 h at 37°C) using 1 µg [^13^C_8_]HDA as internal standard. Cellular lipids were extracted using two consecutive extractions (30 min at RT) with 2 ml of hexane/isopropanol (3∶2, v/v) on a rotary shaker (1,000 rpm). Lipids from supernatants were extracted twice in hexane/methanol (5∶1; v/v, 2 ml). Free FALDs were determined in a similar manner but using PBS (pH 7.4) instead of 1 M HCl. For isolation of free FALD, lipids (from cells and corresponding supernatants) were separated on silica gel 60 plates using hexane/diethyl ether (90∶10, v/v) as the mobile phase. Fractions comigrating with HDA were scraped off and extracted from the TLC sorbent using hexane/diethyl ether (90∶10, v/v). Pentafluorobenzyl (PFB) oximes were prepared from combined lipid extracts (from cells and corresponding supernatants), and (α-chloro) FALDs were quantified by NICI-GC-MS using 2-Cl [^13^C_8_]HDA or [^13^C_8_]HDA as internal standards. Plasmalogen-derived FALDs were calculated by subtracting free from total FALD concentrations. FALD concentrations were converted to plasmalogen concentrations assuming a molar ratio of 1∶1.

### ZO-1 and VE-cadherin Staining

BMVEC were cultured on Permanox chamber slides to confluence. The cells were treated with vehicle or 2-ClHDA at the indicated concentrations for 3 h. After treatment slides were rinsed twice with PBS, dried for 1 to 2 h at RT, and stored at −20°C until required. Cells were fixed in acetone for 5 min at RT, and rehydrated in PBS for 5 min. After blocking nonspecific adsorption with UV ultra block, BMVEC were sequentially incubated for 60 min at RT with anti-human VE-cadherin IgG and rabbit anti-human ZO-1 IgG (diluted 1∶200 and 1∶50 with antibody diluent). Cy-2 labeled goat anti-mouse IgG or Cy-5 labeled goat anti-rabbit IgG (1∶300) were used as secondary antibodies. Between antibody incubation and before mounting with Moviol, samples were rinsed 3 times in PBS for 5 min at RT. Slides were analyzed on a confocal laser-scanning microscope (Leica SP2; Ex/Em = 488/500–535 nm and 647/665–750 nm for Cy-2 and Cy-5, respectively).

### *In situ* Brain Perfusion of 2-ClHDA

Rats were perfused via the common carotid arteries and the magnitude of permeability changes was assessed using sodium fluorescein (SF) as low, and Evans Blue (EB) albumin as a high molecular weight marker. Sprague-Dawley rats were anesthetized with 50 mg/kg pentobarbital. During anesthesia body temperature was maintained at 37°C using a heating panel. After endotracheal intubation the right common carotid artery was exposed and cannulated, whereas the right external carotid artery and the left common carotid artery were ligated. After sectioning of jugular veins animals were perfused for 5 min with oxygenated Ringer solution (supplemented with 18 g/l BSA) at a flow rate of 3 ml/min/hemisphere using a peristaltic pump. Subsequently, perfusion was switched for 90 min to Ringer solution containing 0.4% DMSO (vehicle) or 25 µM 2-ClHDA. For the assessment of BBB function, perfusion with Ringer solution supplemented with SF (1 g/l) and EB (1 g/l, mixed the night before to allow maximal binding of the dye to albumin) was continued for 5 min, followed by washout with dye-free Ringer solution for 7 min. Animals were decapitated, the brains were immediately removed, and cerebral hemispheres were dissected. Brain hemispheres were mechanically homogenized in 3 ml 7.5% (w/v) trichloroacetic acid, and the resulting suspension was neutralized with 5 M NaOH; SF fluorescence (Ex/Em = 484/540 nm) was measured on a Victor 1420 multilabel counter.

### Western Blot Analysis

Total cellular proteins of BMVEC, which were incubated in the absence or presence of NaOCl 2-ClHDA, or PD098059, were separated by SDS-PAGE and electrophoretically transferred to PVDF membranes. Phosphospecific antibodies (rabbit) against mouse p-ERK1/2, p-p38, and p-JNK1/2 (diluted 1∶500; 3% BSA in TBS) were applied by overnight incubation at 4°C. Immunoreactive bands were visualized using HRP-conjugated goat anti-rabbit IgG (1∶5000 in 5% (w/v) nonfat milk powder in TBS-T, 2 h, RT) and subsequent ECL Plus development. For normalization, membranes were stripped at 50°C for 30 min with gentle shaking and reprobed with primary antibodies against the corresponding non-phosphorylated proteins (overnight at 4°C, diluted 1∶1000, 5% (w/v) nonfat milk in TBS). Detection of immunoreactive bands was performed as mentioned above with HRP-conjugated goat anti-rabbit or goat anti-mouse secondary antibodies (1∶5000 or 1∶2500 in 5% (w/v) nonfat milk powder in TBS-T, 2 h, RT) using the ECL system. Immunoreactive bands were quantified by densitometry of films with a HeroLab Easy RH densitometer (HeroLab, Wiesloch, Germany) and EasyWin 32 software.

### Statistical Analyses

Data are presented as means ± SD. To test differences in groups, statistical significance was determined by Student’s t-test or one- or two-way ANOVA with Bonferroni correction (using the GraphPad 5.0 Prism package) as indicated. All values of p≤0.05 were considered significant. *p<0.05, **p<0.01, ***p<0.001.

## Results

### Systemic LPS Induces Neutrophil Accumulation and MPO Release in the Brain Microvasculature

**Figure 1:**
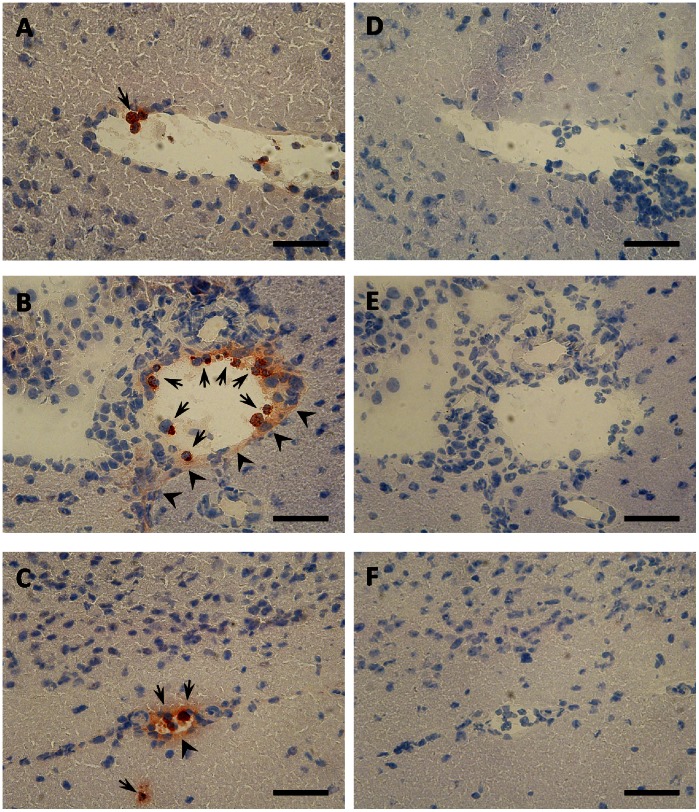
Systemic inflammation induces cell-associated and extracellular (secreted) MPO. C57BL/6 mice received (A, D) PBS or (B, C, E, F) a single systemic LPS injection (250 µg LPS/30 g body weight; i.p.). After 6 h, animals were killed by cervical dislocation, brains were removed and snap frozen in liquid nitrogen. (A-C) Immunostaining of MPO was performed on sagittal cryosections (5 µm) of brain tissue using rabbit anti-human MPO IgG (1∶500) and HRP-labeled goat-anti rabbit IgG (1∶300) as primary and secondary antibodies. MPO (red) was visualized using the AEC system. Staining in a venule (B) and a smaller vessel (C) is shown. (D-F) Negative controls using rabbit non-immune IgG as primary antibody. Sections were counterstained with Mayer's hemalum. Arrows indicate cell-associated MPO, arrowheads indicate secreted MPO located at the abluminal side of vessels. Scale bars: 50 µm.

In the first set of experiments neutrophil accumulation in brain cryosections obtained from control (PBS) and LPS-exposed wild-type mice was studied. The LPS concentration used in the present study (250 µg/30 g body weight) induced a severe acute inflammatory response. Only few MPO-positive cells were observed in venule-like structures of PBS-injected mice (Fig. 1A). In contrast, LPS-treatment resulted in enhanced recruitment of MPO-positive leukocytes in venules (Fig. 1B, arrows) and smaller vessels (Fig. 1C; arrows). In addition to cell-associated MPO substantial amounts of secreted MPO (diffuse extracellular staining indicative for degranulation) could be detected at the luminal and abluminal side of the vessels (Fig. 1B and C; arrowheads). Abluminal localization of externalized MPO is most probably due to endothelial transcytosis [24]. Omission of the primary antibody (not shown) or replacement with non-immune rabbit IgG eliminated all staining (Fig. 1D–F).

**Figure 2:**
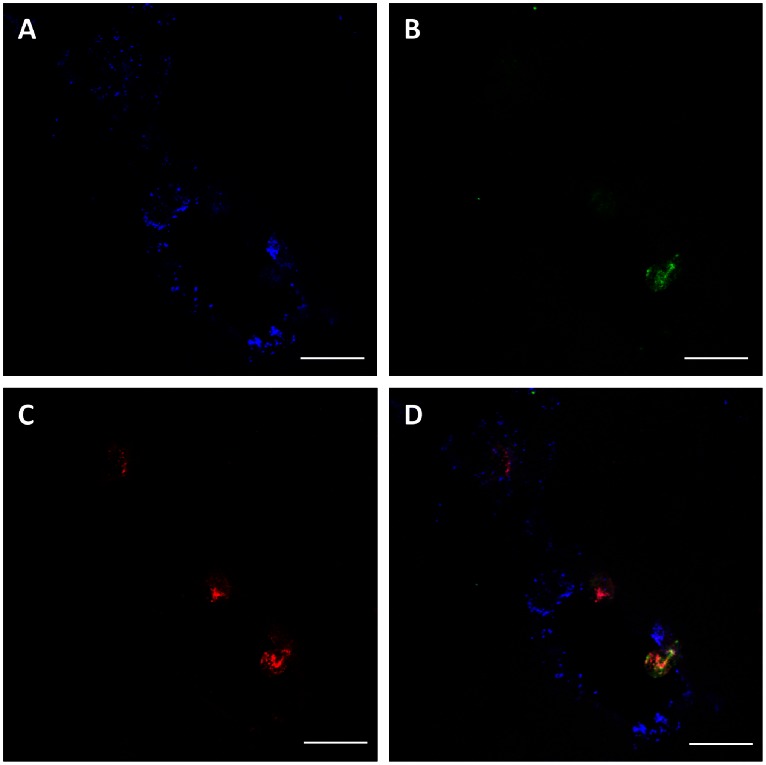
Systemic inflammation induces neutrophil activation and MPO deposition at the cerebrovasculature. C57BL/6 mice received a single systemic LPS injection (250 µg/30 g; i.p.). After 6 h, animals were killed by cervical dislocation, brains were removed and snap frozen in liquid nitrogen. Immunofluorescence staining of (A) endothelial cells, (B) neutrophils and (C) MPO was performed on sagittal cryosections (5 µm) of brain tissue using DyLight 650-labeled rabbit anti-human vWF (1∶75), FITC-labeled rat anti-mouse neutrophil IgG (1∶50) and rabbit anti-human MPO IgG (1∶500) as primary antibodies. Triple immunofluorescence of DyLight 650-labeled anti-human vWF IgG, Cy-3-labeled anti-rabbit IgG and Cy-2-labeled anti-rat IgG was performed by confocal laser scanning microscopy using a Leica SP2. Overlays of blue (vWF), green (neutrophils), and red (MPO) channels are shown in (D). Scale bars: 20 µm.

To corroborate MPO localization at brain endothelial cells, cryosections of LPS-treated mice were stained for vWF, neutrophils, and MPO (Fig. 2). Staining for vWF resulted in a characteristic punctuate staining pattern at the endothelial layer (Fig. 2A) of a vessel containing an adherent and a transmigrating neutrophil (Fig. 2B). MPO was detected in association with neutrophils but also in free form in association with endothelial cells probably resulting from degranulation and/or NETosis [25] (Fig. 2C and D).

### Neutrophil-derived MPO Compromises BMVEC Barrier Function

**Figure 3:**
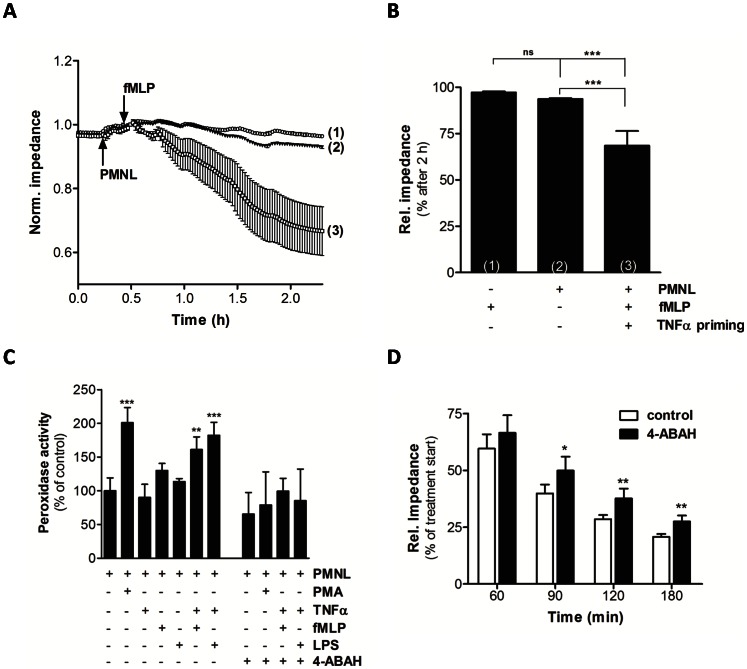
BMVEC barrier function is compromised by activated PMNL. BMVEC were plated on gold microelectrodes and cultured to confluence. Barrier function of hydrocortisone-induced endothelial monolayers (7.5×10^4^ cells) was continuously monitored by impedance sensing at 4 kHz. (A) After stabilization of BMVEC monolayers, fMLP (1), unprimed PMNL (2; 2.5×10^6^), or TNFα-primed/fMLP-activated PMNL (3; 2.5×10^6^) were added to BMVEC monolayers at the indicated time points (arrows). (B) Statistical evaluation of impedance values from (A) after 2 h. Impedance was normalized to baseline recorded before applying the treatments and represents mean values ± SD of four independent experiments (ns = not significant, ***p<0.001, one-way ANOVA).(C) PMNL (2.3×10^6^ cells) were incubated in the presence of a confluent BMVEC (1.5×10^5^) monolayer and stimulated with PMA (250 nM), LPS (1 µg/ml) and fMLP (10 µM) with or without priming with TNFα (10 ng/ml) in the absence or presence of 4-ABAH (100 µM). After 3 h released MPO was determined. Results were normalized to controls (no addition; 100%) and represent means ± SD of triplicate (absence of 4-ABAH) or quadruplicate (presence of 4-ABAH) experiments (**p<0.01, ***p<0.001, one-way ANOVA).(D) After stabilization of BMVEC monolayers induction medium was changed to slightly acidic conditions (HBSS, pH 6). Afterwards, TNFα-primed PMNL were added in the absence or presence of 4-ABAH (100 µM) and activated with fMLP (10 µM). Impedance was normalized to baseline recorded prior to treatments and represents mean values ± SD of four independent experiments (*p<0.05, **p<0.01; Student’s t-test).

In an in vitro approach barrier function of primary porcine BMVEC exposed to non-stimulated or stimulated human PMNL was monitored in real time using the ECIS system (Fig. 3). To mimic inflammatory conditions PMNL were primed with TNFα and stimulated with fMLP. As shown in Fig. 3A and B, fMLP and unstimulated PMNL were without significant effects on barrier function. In contrast, fMLP-mediated activation of TNFα-primed PMNL significantly impaired barrier function. To determine the effects of different stimuli on MPO secretion by PMNL, cells were incubated with PMA, LPS or fMLP. Only PMA induced release of active MPO 2-fold over base line levels (Fig. 3C). When PMNL were primed with TNFα, LPS and fMLP also induced MPO secretion. Under all conditions the MPO inhibitor 4-ABAH reduced peroxidase activity to control levels (Fig. 3C). In line with these results, incubation of BMVEC with stimulated PMNL (TNFα/fMLP) in the presence of 4-ABAH attenuated endothelial barrier dysfunction (Fig. 3D).

**Figure 4:**
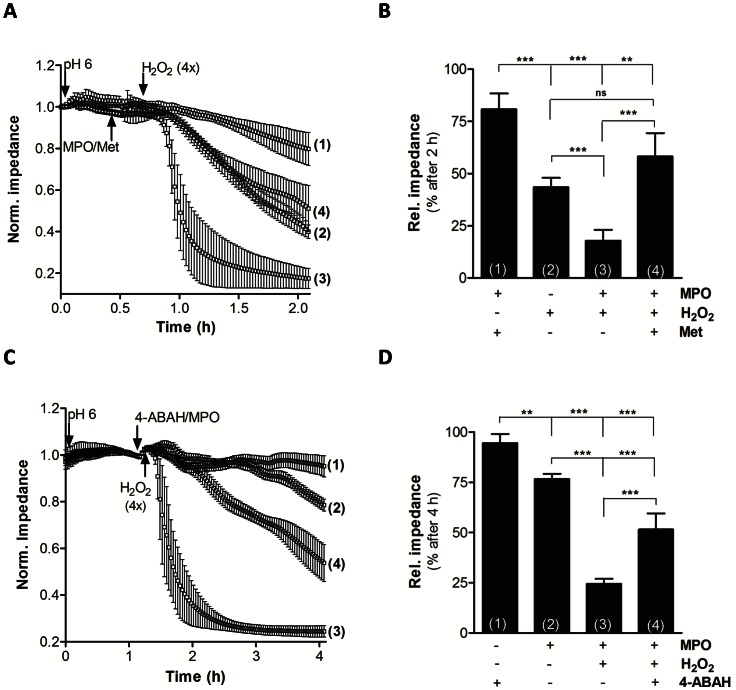
BMVEC barrier function is compromised by the MPO-H_2_O_2_-Cl^−^ system. BMVEC were plated on gold microelectrodes and cultured to confluence. Barrier function of hydrocortisone-induced endothelial monolayers (7.5×10^4^ cells) was continuously monitored by impedance sensing at 4 kHz. (A) After stabilization of BMVEC monolayers induction medium was changed (arrow) to slightly acidic conditions (HBSS, pH 6). After a 30 min pre-conditioning period, BMVEC were incubated (arrow) with MPO (120 nM) and methionine (1; ‘Met’; 5 mM), H_2_O_2_ (2; 4×125 µM every 3 min), MPO and H_2_O_2_ (3), or MPO, H_2_O_2_ and Met (4). (B) Statistical evaluation of impedance values after 2 h from (A). Impedance was normalized to baseline and represents mean values ± SD of 4 independent experiments (ns = not significant; **p<0.01, ***p<0.001, one-way ANOVA). (C) After stabilization of BMVEC monolayers induction medium was changed (arrow) to slightly acidic conditions (HBSS, pH 6). After a 70 min pre-conditioning period, BMVEC were incubated (arrow) with 4-ABAH (1; 100 µM), MPO (2; 120 nM), MPO and H_2_O_2_ (3; 4×125 µM), or MPO, H_2_O_2_ and 4-ABAH (4). (D) Statistical evaluation of impedance values after 4 h from (C). Impedance was normalized to baseline and represents mean values ± SD of 4 independent experiments (**p<0.01, ***p<0.001, one-way ANOVA).

To further investigate the impact of MPO release on barrier function, BMVEC were exposed to either H_2_O_2_ alone or in combination with exogenously added MPO. H_2_O_2_ treatment resulted in a significant decrease of barrier function by 60%, while the presence of MPO and H_2_O_2_ decreased barrier function by 80% (Fig. 4A). Methionine, a potent HOCl scavenger, significantly attenuated barrier dysfunction to levels observed with H_2_O_2_ alone. A statistical evaluation of the impedance data at the 2 h time point is shown in Fig. 4B. As observed with stimulated PMNL (Fig. 3D) a rescue of barrier function was observed when BMVEC were exposed to MPO and H_2_O_2_ in the presence of 4-ABAH (Fig. 4C). MPO added in the absence of H_2_O_2_ decreased barrier function by ≈ 15%. A statistical evaluation of the impedance data at 3 h is shown in Fig 4D.

**Figure 5:**
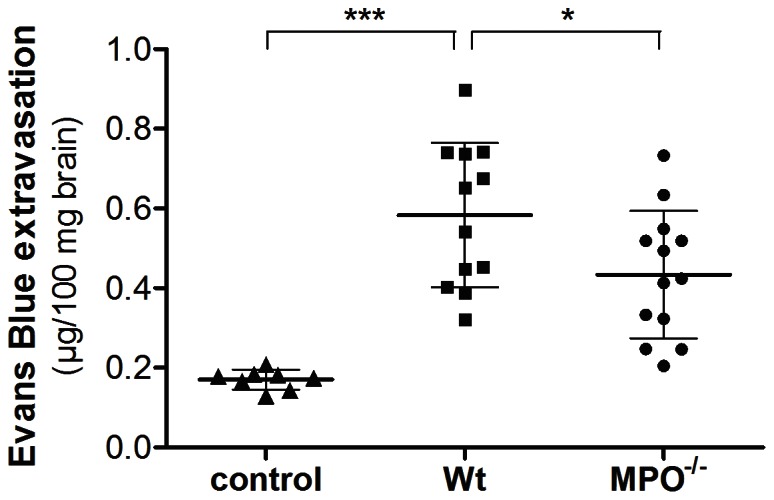
BBB permeability in response to LPS is attenuated in MPO^−/−^ mice. Control animals (‘control’; n = 8) were injected with Evans Blue (EB) in PBS. Wt (n = 12) and MPO^−/−^ mice (MPO^−/−^; n = 13) received a single injection (i.p.) of LPS (250 µg LPS/30 g body weight) and EB. Twelve hours post LPS treatment animals were anaesthetized with pentobarbital (150 mg/kg body weight) and transcardially perfused with 25 ml PBS. Thereafter brains were removed, frozen in liquid nitrogen and homogenized. EB was quantitated spectrophotometrically using an external EB calibration curve. Results shown represent mean values ± SD (*p<0.05; ***p<0.001, one way ANOVA).

To substantiate the contribution of MPO to LPS-induced BBB dysfunction in vivo, EB extravasation was compared in brains of control (PBS)- and LPS-treated wild-type and MPO^−/−^ mice. In response to peripheral LPS, EB extravasation was increased in wild-type and MPO^−/−^ mice (3.4- and 2.5-fold, respectively) in comparison to control (PBS) animals (Fig. 5). The mean EB concentration detected in MPO^−/−^ animals was significantly lower as compared to wild-type littermates (0.43 vs. 0.58 µg/100 mg brain tissue, respectively). These data reveal a quantitatively important contribution of MPO to LPS-induced BBB dysfunction in vivo.

### MPO-generated Chlorinated Aldehydes Impair BBB Function

**Figure 6:**
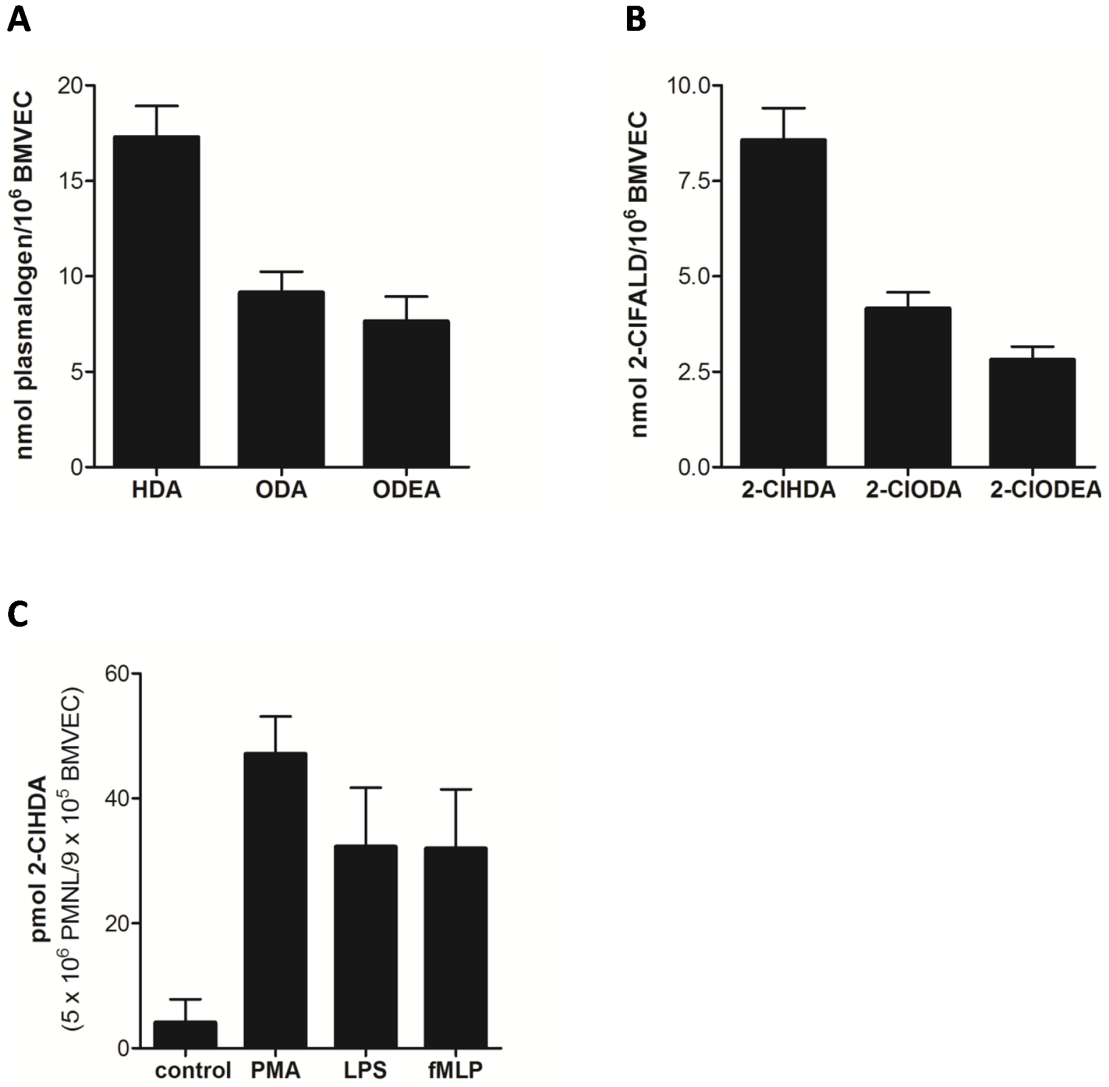
Modification of endogenous BMVEC plasmalogens by the MPO-H_2_O_2_-Cl^−^ system or TNFα-primed/fMLP-activated neutrophils generate chlorinated aldehydes. (A) Adherent BMVEC were hydrolyzed (0.5 M HCl, 37°C, 2 h) in the presence of 1 µg [^13^C_8_]HDA as internal standard. Following extraction, PFB oximes were prepared, and total FALD concentrations (hexadecanal, HDA; octadecanal, ODA; octadecenal, ODEA) were quantitated by NICI-GC-MS analysis. FALD content was converted to plasmalogen concentrations (molar ratio = 1∶1). Results represent mean values ± SD from 3 independent experiments. (B) BMVEC were washed with HBSS (pH 6) and incubated in the presence of MPO (120 nM) and H_2_O_2_ (500 µM). One µg 2-Cl[^13^C_8_]HDA) was added as internal standard at treatment start. 2-chlorohexadecanal (2-ClHDA), 2-chlorooctadecanal (2-ClODA), and 2-chlorooctadecenal (2-ClODEA) were analyzed as PFB-oximes by NICI-GC-MS. Results are shown as mean values ± SD of 2-ClFALD concentrations from 4 independent experiments. (C) BMVEC were washed with HBSS (pH 6) and incubated with PMA or primed with TNFα (10 ng/ml) followed by activation with LPS (1 µg/ml) or fMLP (10 µM) for 3 h. 2-Cl[^13^C_8_]HDA (250 ng) were added as internal standard after treatment start. Lipids were extracted, converted to PFB oximes, and 2-ClHDA concentrations were quantitated by NICI-GC-MS. Results represent mean values ± SD from 3 independent experiments.

Plasmalogens, indispensable lipid components of plasma membranes of mammalian cells are accessible to modification by the MPO-H_2_O_2_-Cl^−^ system. NICI-GC-MS analyses revealed that 1-O-16∶0 (quantitated as hexadecanal; HDA; 17.3 nmol/10^6^ cells) followed by 1-O-18∶0 (octadecanal; ODA; 9.2 nmol/10^6^ cells) and 1-O-18∶1 (octadecenal; ODEA; 7.6 nmol/10^6^ cells) represent the majority of plasmalogen species in BMVEC (Fig. 6A). To investigate whether BMVEC plasmalogens are susceptible to HOCl modification, cells were incubated in the presence of the MPO-H_2_O_2_-Cl^−^ system. Product analysis by NICI-GC-MS revealed the formation of 8.6 nmol/10^6^ cells of 2-ClHDA followed by 2-ClODA (4.2 nmol/10^6^ cells) and 2-ClODEA (2.8 nmol/10^6^ cells) (Fig. 6B). Co-incubation of activated PMNL (PMA, TNFα/LPS, TNFα/fMLP) and BMVEC also induced generation of 2-ClHDA (depending on the stimulus between 30 and 50 pmol 2-ClHDA/10^6^ PMNL; Fig. 6C).

**Figure 7:**
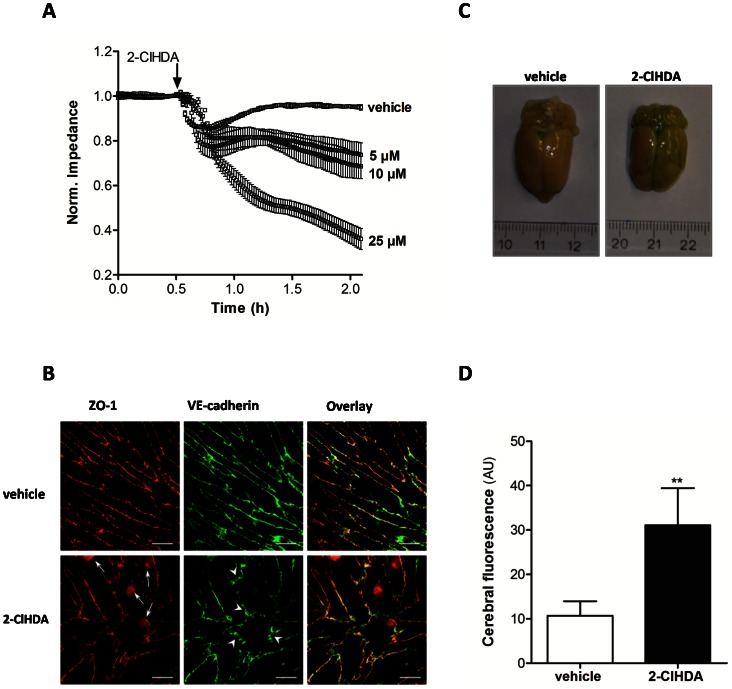
2-ClHDA impairs barrier function in vitro and in vivo. (A) BMVEC were plated on gold microelectrodes and cultured to confluence. Barrier function of HC-induced BMVEC was continuously monitored by impedance sensing at 4 kHz in the presence of the indicated 2-ClHDA concentrations or vehicle (DMSO). (B) BMVEC were cultured on coverslips until confluence. After incubation for 3 h in the presence of vehicle (DMSO; upper panel) or 15 µM 2-ClHDA (lower panel) immunofluorescence labeling of ZO-1 (red) and VE-cadherin (green) was performed. Sites of nuclear ZO-1 redistribution (arrows) and frizzy-like structures (arrowheads) are indicated. Scale bars: 20 µm. (C, D) The left common carotid artery of anesthetized rats was exposed and cannulated. After sectioning of jugular veins animals were perfused (3 ml/min) with Ringer solution for 5 min. Subsequently, perfusion was switched for 90 min to Ringer solution containing vehicle (DMSO) or 25 µM 2-ClHDA. This was followed by perfusion with Ringer solution supplemented with EB and SF for 5 min and a washout with Ringer solution (without dyes) for 7 min. Animals were decapitated and the brains were immediately removed. (C) Macroscopic evaluation of EB extravasation, and (D) determination of SF fluorescence intensity in brain homogenates are shown. Results represent mean values ± SD from 4 animals (**p<0.01, Students t-test).

Next we extended our recent studies [19] and investigated concentration-dependent effects of 2-ClHDA on barrier function. We now show that 2-ClHDA increases permeability in a concentration-dependent manner with 5 µM appearing to be the threshold concentration (Fig. 7A). At 25 µM 2-ClHDA induced almost complete loss of barrier function while concentrations <5 µM were without effect (data not shown).

To reveal possible rearrangement of junctional complexes in response to 2-ClHDA, ZO-1 (tight junctions) and VE-cadherin (adherens junctions) morphology was examined by immunofluorescence microscopy (Fig. 7B). BMVEC were incubated either with vehicle (upper panel) or with 2-ClHDA (lower panel). In comparison to vehicle-treated cells 2-ClHDA induced two major morphological alterations: First, 2-ClHDA-mediated translocation of originally junction-associated ZO-1 into the nucleus (arrows). Second, VE-cadherin staining revealed that junctional strands turned into ‘frizzy’-like structures (arrowheads) which indicates impaired junctional interaction (Fig. 7B).

To corroborate 2-ClHDA-mediated BBB dysfunction in vivo, rat brain perfusion experiments were performed. Macroscopic analysis revealed pronounced extravasation of the high molecular weight marker EB-albumin in response to perfusion of 2-ClHDA (Fig. 7C). In line, the accumulation of SF (a low molecular weight permeability marker) was significantly higher (3-fold) in 2-ClHDA-perfused hemispheres (Fig. 7D). These findings demonstrate that 2-ClHDA increases BBB permeability towards high and low molecular weight compounds.

### ERK1/2 and JNK1/2 are Involved in 2-ClHDA-induced Barrier Dysfunction

**Figure 8:**
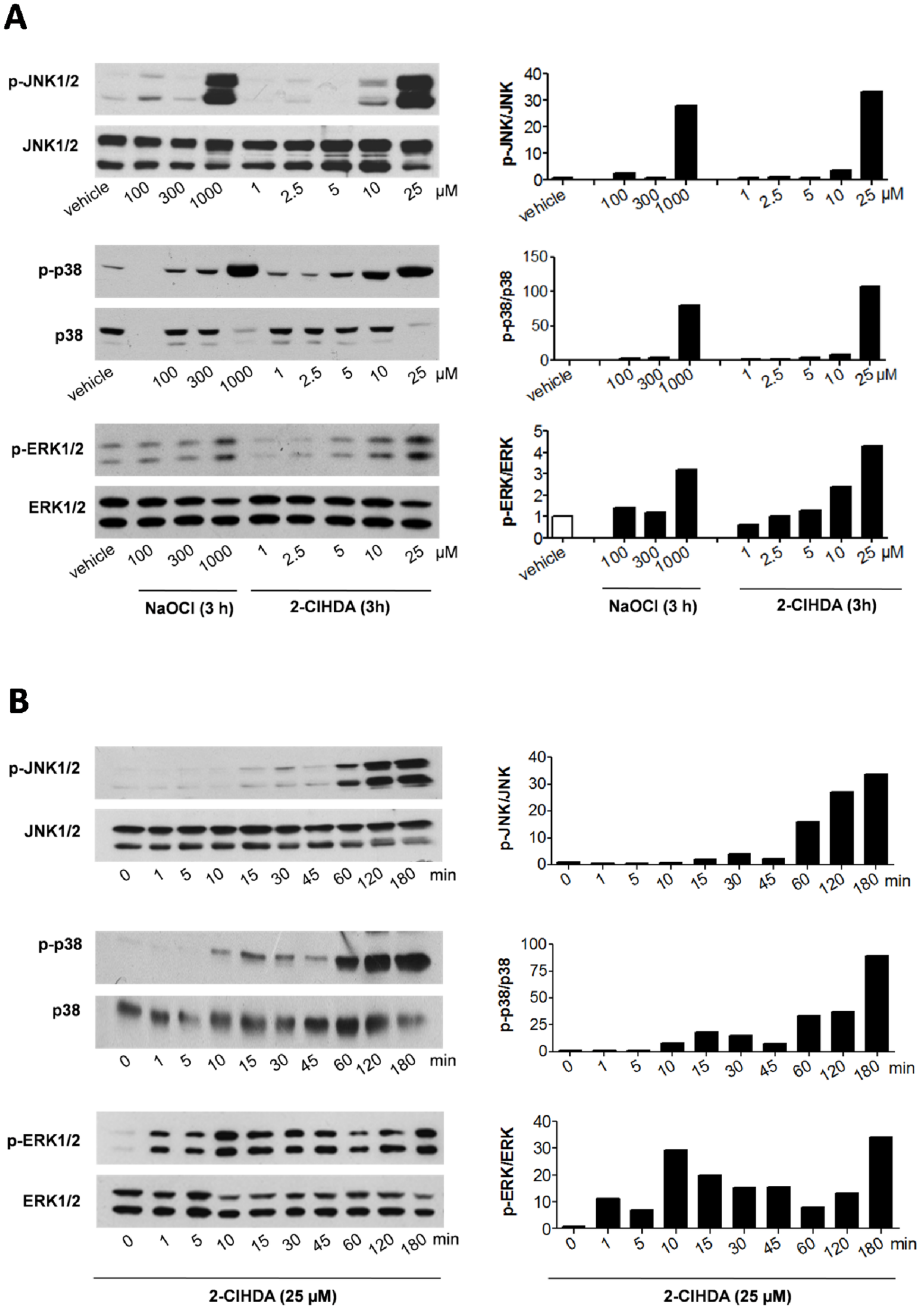
2-ClHDA activates MAPK pathways. (A) Concentration-dependent activation of the MAPK cascade. BMVEC were incubated with NaOCl or 2-ClHDA or DMSO (used as vehicle for 2-ClHDA delivery) at the indicated concentrations for 3 h. (B) Time-dependent activation of the MAPK cascade in response to 2-ClHDA. BMVEC were incubated with 2-ClHDA (25 µM) for the indicated time periods. After treatment, cells were lysed, aliquots of protein lysates were subjected to SDS-PAGE and transferred to PVDF membranes. Pan- or phospho-specific polyclonal antibodies against p38, JNK1/2, or ERK1/2 were used as primary antibodies. Immunoreactive bands were visualized with peroxidase-conjugated secondary antibodies using the ECL-system. Bar graphs in the right panels show the ratio of optical densities of immunoreactive phosphorylated normalized to non-phosphorylated proteins.

Signaling events mediated by ERK1/2 and stress-activated protein kinases (JNK1/2 and p38) play a critical role in the regulation of barrier integrity [26], [27]. To gain insight into 2-ClHDA-induced downstream signaling, concentration- and time-dependent MAPK activation was studied. In comparison to NaOCl (activating MAPK signaling at 300–1000 µM; Fig. 8A), 2-ClHDA activated the MAPK pathway at much lower concentrations. JNK1/2 was activated at 2-ClHDA concentrations of ≥10 µM, p38 and ERK1/2 were activated at even lower concentrations (Fig. 8A). JNK and p38 activation revealed a first peak between 15 and 60 min, followed by a substantially more pronounced activation between 60 and 180 min (Fig. 8B). In contrast to the rather slow activation of stress-activated protein kinases, p-ERK1/2 was induced within 1 min post 2-ClHDA addition and remained activated up to 3 h (Fig. 8B). The bar graphs (Fig. 8; right panels) show densitometric evaluation of Western blots and display the ratio of optical densities of phosphorylated normalized to non-phosphorylated proteins.

**Figure 9:**
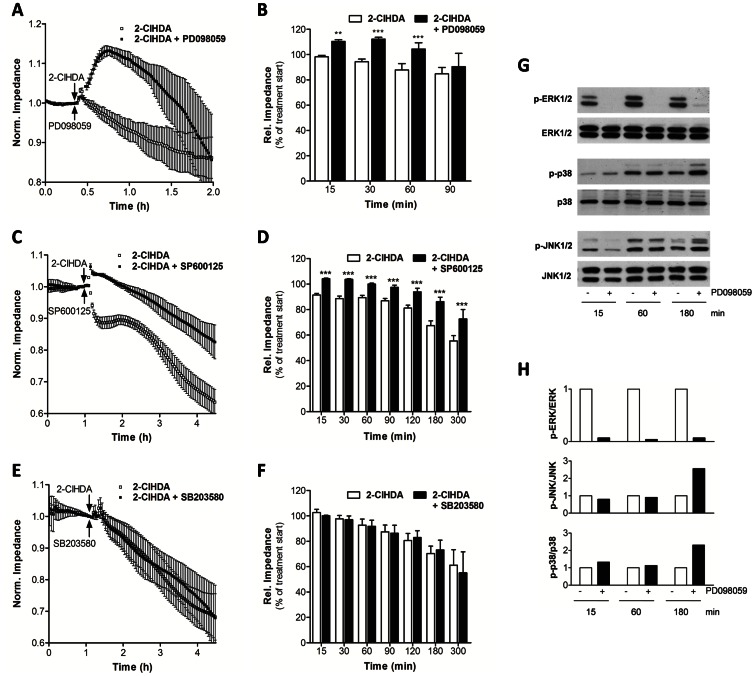
Inhibition of ERK and JNK provides partial rescue against 2-ClHDA-induced barrier dysfunction. BMVEC were plated on gold microelectrodes and cultured to confluence. Barrier function of endothelial monolayers was continuously monitored by impedance sensing at 4 kHz. After stabilization, cells were challenged (arrow) with 2-ClHDA in the absence or presence of (A) 100 µM PD098059, (C) 25 µM SP600125, or (E) 25 µM SB203580. Results represent mean values ± SD from 4 independent experiments. 2-ClHDA concentrations were 5 (A) and 10 (C, E) µM. (B, D, and F) Statistical evaluation of relative barrier function at the indicated time periods post 2-ClHDA treatment in the absence or presence of the respective antagonist. Impedance was normalized to baseline and represent mean values ± SD of 4 independent experiments (**p<0.01; ***p<0.001; two-way ANOVA). (G) BMVEC were incubated with 2-ClHDA (25 µM) in the absence or presence of PD098059 (100 µM) for the times indicated. Cells were lysed, aliquots of protein lysates were subjected to SDS-PAGE and transferred to PVDF membranes. (Phospho)Specific polyclonal antibodies against ERK1/2, p38, or JNK1/2 were used as primary antibodies. Immunoreactive bands were visualized with peroxidase-conjugated secondary antibodies using the ECL-system. (H) Bar graphs represent the ratio of optical densities of immunoreactive phosphorylated proteins normalized to non-phosphorylated proteins.

Prompted by these observations we investigated the effects of pharmacological MAPK antagonists on 2-ClHDA-mediated barrier dysfunction in vitro. Cells were challenged with 2-ClHDA and MAPK signaling was inhibited by PD098059 (a MAPK kinase inhibitor that prevents ERK1/2 activation; 100 µM), SP600125 (JNK1/2 inhibitor, 25 µM), or SB203580 (p38 inhibitor, 25 µM). ECIS analysis revealed transient barrier rescue by PD098059 (Fig. 9A and B), long-term protection by SP600125 (Fig. 9C and D), while SB203580 was without effects on 2-ClHDA-induced BMVEC barrier dysfunction (Fig. 9E and F). Since the PD098059 concentration used during the ECIS experiments was high (and known to impact on activation of other members of the MAPK family; Ref. [28]) we evaluated activation of JNK1/2 and p38 MAPK by Western blotting. As expected, 2-ClHDA-induced ERK1/2 activation was completely blocked by PD098059 (Fig. 9G). In contrast, JNK1/2 and p38 phosphorylation was approx. 2.5-fold increased in the presence of 100 µM PD098059 at the 180 min time point (Fig. 9G). The bar graphs (Fig. 9H) show densitometric evaluation of Western blots and display the ratio of optical density of phosphorylated normalized to non-phosphorylated proteins.

## Discussion

BBB dysfunction is commonly observed during sepsis and contributes to neuronal dysfunction. We examined the consequences of LPS-induced severe systemic inflammation on neutrophil accumulation and MPO release at the cerebrovasculature and investigated the role of MPO and MPO-derived oxidants on BBB function in vitro and in vivo.

Our data show that neutrophils adhere to the cerebrovasculature in a mouse model of LPS-induced systemic inflammation and release/deposit MPO that does not colocalize with neutrophils (Fig. 1 and 2). Comparable findings of MPO release from neutrophils and macrophages/microglia were reported in a mouse model of stroke [29]. Whether this is due to NETosis was not investigated in the present study. NET formation depends on MPO [25] and HOCl [30] and is inhibited by the HOCl scavenger taurine [30]. NETs are able to induce endothelial dysfunction [15] and NET-associated MPO retains its activity [31]. Therefore released MPO might have the capacity to modify BMVEC lipids and/or proteins, thereby contributing to BBB dysfunction. One potential candidate producing H_2_O_2_ as cosubstrate for HOCl production in the cerebrovascular endothelium is NADPH oxidase 4 [32]. This enzyme shows highest expression in the cerebrovasculature and in neurons [33]. Our data support such a mechanism: Both, enzymatically active MPO and fMLP-stimulated neutrophils induced barrier dysfunction of primary BMVEC in vitro. This increase in permeability was partially reversed by the MPO inhibitor 4-ABAH and the HOCl scavenger methionine (Fig. 3 and 4). Of note, LPS-induced BBB dysfunction was significantly lower in MPO^−/−^ mice as compared to their wild-type littermates. The MPO-dependent component of BBB dysfunction contributes to approx. 25% (Fig. 5). Several independent studies revealed beneficial effects of MPO knockdown on disease progression: Deletion of MPO resulted in decreased loss of neurons in the substantia nigra of MPO^−/−^ mice in response to the Parkinsonian agent methyl-phenyl-tetrahydropyridine [34], attenuated LPS-induced acute lung inflammation [35], and preserved expression of TJ-associated claudins in lungs of influenza-infected mice [36]. Pharmacological inhibition of MPO reduced the severity of clinical symptoms and tissue damage in brain and improved survival in a mouse model of MS [11] and multiple system atrophy [37]. On the other hand, astrocyte-specific overexpression of human MPO in an Alzheimer’s disease mouse model resulted in cognitive decline, accumulation of the lipid peroxidation product 4-hydroxynonenal along with the formation of phospholipid- and plasmalogen-derived hydro(pero)xides [38]. Mouse neutrophil MPO activity is about 5 times lower than that of the human neutrophil enzyme [39]. Considering the prevalent occurrence of vessel wall-associated MPO in human diseases [40], MPO-mediated reactions are expected to provide a significant contribution to disease progression and oxidative tissue damage [41]. Notwithstanding, neutrophils from MPO^−/−^ mice show altered cytokine and chemokine production [35] that can affect BBB function. This is an important aspect determining the outcome of an inflammatory response that was not addressed during the present study.

Plasmalogens represent a major and essential phospholipid class in the brain that is susceptible towards HOCl modification. During one of our earlier studies we demonstrated that peripherally induced inflammation results in a significant decrease of brain plasmalogen concentrations [16]. In agreement with data reported for human coronary artery endothelial cells [42], we detected high plasmalogen concentrations in BMVEC during the present study. Moreover, part of the endogenous plasmalogen pool was converted to 2-ClFALD (Fig. 6).

HOCl modification of plasmalogens is probably one of the key reactions during MPO-mediated BBB compromise: First, plasmalogens are enriched in lipid raft domains, which are structural determinants for correct junctional positioning at the BBB [17], [43]. The rate constants for HOCl-dependent plasmalogen modification are approx. 10-fold higher than that of non-vinylether containing phospholipids [44]. Presumably, plasmalogen modification by MPO-derived HOCl would alter the BMVEC lipid environment thereby interfering with junctional patterning (Fig. 7B) and BBB function.

Second, micromolar concentrations of 2-ClHDA (undetectable in brains of MPO^−/−^ mice) that are generated under inflammatory conditions in vivo [16], [45], [46] were sufficient to cause significant BBB breakdown in vitro and in vivo (Fig. 7). One plausible explanation for these observations is the ability of 2-ClHDA to form covalent adducts with lysine residues in proteins via Schiff base formation [47]. These events would affect barrier function if junctional proteins, vital transport proteins or proteins of the extracellular matrix were subject to 2-ClHDA-mediated modification. Treatment of BMVEC with 2-ClHDA induced morphological alterations: A change from continuous distribution of junctional proteins to ‘frizzy-like’ structures and the transformation from spindle to a more rounded cell shape were identified. Frizzy-like junctional architecture can be induced by stress-related TJ disassembly, an observation that is in line with our findings of ZO-1 accumulation in the nuclear compartment (Fig. 7B). Translocation of ZO-1 to the nucleus was previously reported [48] and evidence exists that ZO-1/ZONAB regulates G_1_/S-phase transition [49].

Third, results from the present study show that 2-ClHDA can elicit a stress signaling response in BMVEC (Fig. 8). We have recently demonstrated [19] that 2-ClHDA activates procaspase 3 and induces cellular ATP depletion. Of note, activation of MAPK pathways under conditions of oxidative stress or inflammatory conditions is associated with barrier dysfunction [27], [48]. Rapid activation of ERK1/2 in response to 2-ClHDA identifies this pathway as an early effector of aldehyde stress-induced barrier dysfunction in endothelial cells [50]. ERK1/2-phosphorylation is associated with increased expression of matrix metalloproteinase (MMP)-9 and proteolytic cleavage of TJ complexes during focal cerebral ischemia [51], [52]. Whether or not 2-ClHDA-mediated breakdown of BBB function in vivo (Fig. 5) involves MMP-mediated degradation of the extracellular matrix and subsequent detachment of perivascular cells [53] remains to be elucidated. H_2_O_2_-induced alterations in TJ architecture were prevented by ERK1/2 antagonism with the MAPK kinase inhibitor PD098059 [54]. In line, our data revealed that ERK1/2 antagonism by PD098059 provided transient protection against 2-ClHDA-induced barrier dysfunction in vitro (Fig. 9A and B). Whether this transient effect could be due to upregulation of JNK and p38 by PD098059 (100 µM during the present study) is currently not clear. The JNK antagonist SP600125 provided protection against 2-ClHDA-induced barrier dysfunction over longer times (5 h, Fig. 9C and D), while the p38 inhibitor SB203580 was without effect.

Pharmacological antagonism of p38 and JNK1/2 activation attenuates BBB disruption in different animal models. In a rat model of transient focal ischemia the p38 inhibitor SB203580 significantly attenuated BBB dysfunction [55]. In a rat middle cerebral artery occlusion model of ischemic stroke the p38 and JNK inhibitors SB239063 and SP600125 reduced brain damage and hemorrhage [56]. The complex roles of JNK1/2 for acute brain injury have been investigated in models of subarachnoid hemorrhage and transient focal cerebral ischemia: Inhibition of JNK1/2 protects against mitochondrial apoptosis, reduces MMP-9 levels, inhibits BBB disruption, and reduces edema formation [57], [58]. Despite evidence for beneficial effects of p38 MAPK inhibition on loss of BBB integrity, pharmacological antagonism was without effect on 2-ClHDA-induced barrier loss during the present study (Fig. 9E and F). The reason for this observation is currently unclear.

In summary, the effects of MPO during LPS-mediated BBB breakdown involve multiple pathways, including the release of HOCl, formation of cytotoxic and signaling-active compounds like 2-ClHDA, or indirectly by increasing inflammatory cell recruitment via electrostatic interaction of MPO with the brain endothelium thereby recruiting additional neutrophils [59]. Application of MPO inhibitors [60] might therefore represent an attractive strategy to interfere with BBB breakdown in sepsis patients.
